# Acute Effects of Fine Particulate Air Pollution on Cardiac Arrhythmia: The APACR Study

**DOI:** 10.1289/ehp.1002640

**Published:** 2011-03-11

**Authors:** Fan He, Michele L. Shaffer, Sol Rodriguez-Colon, Jeff D. Yanosky, Edward Bixler, Wayne E. Cascio, Duanping Liao

**Affiliations:** 1 Department of Public Health Sciences and; 2 Sleep Research and Treatment Center, Department of Psychiatry, the Pennsylvania State University College of Medicine, Hershey, Pennsylvania, USA; 3 Department of Cardiovascular Sciences, Brody School of Medicine, and the East Carolina Heart Institute at East Carolina University, Greenville, North Carolina, USA

**Keywords:** cardiac arrhythmia, cardiovascular disease, PAC, particulate matter, PVC

## Abstract

Background: The mechanisms underlying the relationship between particulate matter (PM) air pollution and cardiac disease are not fully understood.

Objectives: We examined the effects and time course of exposure to fine PM [aerodynamic diameter ≤ 2.5 μm (PM_2.5_)] on cardiac arrhythmia in 105 middle-age community-dwelling healthy nonsmokers in central Pennsylvania.

Methods: The 24-hr beat-to-beat electrocardiography data were obtained using a high-resolution Holter system. After visually identifying and removing artifacts, we summarized the total number of premature ventricular contractions (PVCs) and premature atrial contractions (PACs) for each 30-min segment. A personal PM_2.5_ nephelometer was used to measure individual-level real-time PM_2.5_ exposures for 24 hr. We averaged these data to obtain 30-min average time–specific PM_2.5_ exposures. Distributed lag models under the framework of negative binomial regression and generalized estimating equations were used to estimate the rate ratio between 10-μg/m^3^ increases in average PM_2.5_ over 30-min intervals and ectopy counts.

Results: The mean ± SD age of participants was 56 ± 8 years, with 40% male and 73% non-Hispanic white. The 30-min mean ± SD for PM_2.5_ exposure was 13 ± 22 μg/m^3^, and PAC and PVC counts were 0.92 ± 4.94 and 1.22 ± 7.18. Increases of 10 μg/m^3^ in average PM_2.5_ concentrations during the same 30 min or the previous 30 min were associated with 8% and 3% increases in average PVC counts, respectively. PM_2.5_ was not significantly associated with PAC count.

Conclusion: PM_2.5_ exposure within approximately 60 min was associated with increased PVC counts in healthy individuals.

Numerous epidemiological studies have demonstrated a consistent link between particulate matter (PM) air pollution, especially fine PM [aerodynamic diameter ≤ 2.5 μm (PM_2.5_)], and increased cardiopulmonary morbidity and mortality (Brook et al. 2004; Mann et al. 2002; Pope et al. 2002; Samet et al. 2009; Schwartz 1999). Previous studies have suggested that PM_2.5_ exposure is significantly associated with increased heart rate and decreased heart rate variability (HRV; Gold et al., 2000; He et al. 2010; Liao et al. 1999; Luttmann-Gibson et al. 2006; Magari et al. 2001; Park et al. 2005). Increased heart rate and impaired HRV are indicative of autonomic nervous system dysfunction in the direction of increased sympathetic outflow and weakened parasympathetic modulation. Such an impaired autonomic modulation may decrease the threshold of arrhythmia and trigger the onset of premature ventricular contractions (PVCs) and premature atrial contractions (PACs) (Huikuri et al. 2001), the most common forms of arrhythmia in the general population (Engel et al. 2007; Rautaharju et al. 2006). It is therefore biologically plausible that higher exposures to PM_2.5_ may increase the risk of common forms of arrhythmia. Most of the previous studies relating air pollution exposure and arrhythmia were carried out in patients with implantable cardioverter defibrillators (ICDs; Dockery et al. 2005; Peters et al. 2000; Vedal et al. 2004). Patients with ICDs often have severe underlying structural cardiovascular conditions. Thus, the results from such patient-based data may not reflect the effect of PM_2.5_ exposure on arrhythmia in the general population, especially among healthy persons. Recently, Liao et al. (2009) reported that elevated geographic information systems–estimated ambient PM_2.5_ exposures 1 day before the electrocardiography (ECG) measurement were associated with increased odds of PVC in women enrolled in the Women’s Health Initiative (WHI) clinical trials study. However, very little is known about arrhythmic effects of individual-level PM_2.5_ exposures or the time course of PM_2.5_ effects.

We designed this study to investigate the effects and the time course, that is, the time period from exposure to cardiac response, of individual-level PM_2.5_ exposures on the risk of PVC and PAC from 24-hr beat-to-beat Holter ECG recordings in a middle-age community-dwelling sample of healthy nonsmokers.

## Materials and Methods

*Population.* For this report, we used the data collected for the Air Pollution and Cardiac Risk and Its Time Course (APACR) study, which we designed to investigate the mechanisms and time course of the adverse effects of PM_2.5_ on cardiac electrophysiology, blood coagulation, and systemic inflammation. Recruitment methods and examination procedures for the APACR study have been published elsewhere (He et al. 2010; Liao et al. 2010). Briefly, all study participants were recruited from communities in central Pennsylvania, primarily from the Harrisburg metropolitan area. The inclusion criteria for the study included nonsmoking adults ≥ 45 years old who had not been diagnosed with severe cardiac problems (defined as diagnosed valvular heart disease, congenital heart disease, acute myocardial infarction or stroke within 6 months, or congestive heart failure). Approximately 75% of the individuals who were contacted and who met our inclusion criteria were enrolled in the APACR study. Our targeted sample size was 100 individuals, and we enrolled and examined 106 individuals for the APACR study. One participant was excluded because of extremely high-frequency and complex PVCs. PM_2.5_ and Holter ECG monitoring was initiated between 0800 and 1000 hours on day 1, immediately after participants were administered a standardized questionnaire and underwent a brief physical examination and a fasting blood draw by a trained and certified research nurse. The next morning, the participants returned to remove the monitors and had their second blood draw. The APACR study has maintained approval by the Pennslyvania State University College of Medicine institutional review board. All participants gave written informed consent before their participation in the study.

*Personal PM_2.5_ exposures.* The APACR study used a personal PM_2.5_ DataRam (pDR, model 1200; Thermo Scientific, Boston, MA) for real-time 24-hr personal PM_2.5_ exposure assessment. Details of the exposure assessment (He et al. 2010; Liao et al. 2010) and of the instrument’s performance have been reported elsewhere (Rea et al. 2001; Reed et al. 2000; Wallace et al. 2005; Williams et al. 2009). Real-time PM_2.5_ concentrations were recorded at 1-min intervals and averaged over 30-min segments, beginning on the hour or half-hour. Therefore, the PM_2.5_ exposure variables were treated as repeated measures, and each individual contributed 48 exposure data points over 24 hr.

*Continuous ambulatory ECG and arrhythmia.* A high-fidelity (sampling frequency, 1,000 Hz) 12-lead HScribe Holter System (Mortara Instrument, Inc., Milwaukee, WI) was used to collect the 24-hr Holter beat-to-beat ECG data. The details of the Holter ECG data collection and reading have been published elsewhere (He et al. 2010; Liao et al. 2010). Relevant to this report, the Holter ECG data were scanned to a designated computer for offline processing by an experienced investigator using HScribe System software (version 4.21; Mortara Instrument, Inc.). The main objective of the offline processing was to verify the Holter-identified ECG waves and to identify and label additional electronic artifacts and arrhythmic beats in the ECG recording. An experienced ECG reader visually reviewed and verified all arrhythmic beats in the ECG recording. For each participant, we calculated the 30-min segment-specific PVC and PAC counts and summed the PVC and PAC counts to determine the total ectopic beats. The counts were treated as repeated measures, and each individual contributed 48 data points for each of the three arrhythmic beat counts to the analysis. We performed time- and frequency-domain HRV analysis on the artifact-free and ectopy-free normal R-to-R intervals on each of the 30-min–based ECG segments, according to current recommended practice (Task Force of the European Society of Cardiology 1996). The following HRV indices were used as indices of cardiac autonomic modulation: standard deviation of all R-to-R intervals (SDNN; milliseconds), square root of the mean of the sum of the squares of differences between adjacent R-to-R intervals (RMSSD; milliseconds), power in the low-frequency (LF; 0.04–0.15 Hz) and high-frequency (HF; 0.15–0.40 Hz) range, and the ratio of LF and HF.

*Other individual-level covariables.* A standardized questionnaire developed by APACR investigators was used to collect the following individual-level information: demographic variables, including age, race, sex, and highest education level; medication uses, including antianginal medication, antihypertensive medication, and antidiabetic medication; and physician-diagnosed chronic disease history, including cardiovascular disease (CVD; revascularization procedures and myocardial infarction), hypertension, and diabetes. The averages of the second and third measures of seated systolic and diastolic blood pressures on day 1 were used to represent a participant’s blood pressure levels. Day 1 fasting glucose was measured by the Pennsylvania State University College of Medicine General Clinical Research Center’s central laboratory (Hershey, PA). CVD was defined by antianginal medication use or a history of CVD. Hypertension was defined by antihypertensive medication use, physician-diagnosed hypertension, systolic blood pressure ≥ 140 mmHg, or diastolic blood pressure ≥ 90 mmHg. Diabetes was defined by antidiabetic medication use, physician-diagnosed diabetes, or fasting glucose > 126 mg/dL. Body mass index (BMI) was defined as the ratio of weight to height squared.

*Weather variables.* We obtained real-time temperature and relative humidity using a HOBO H8 logger (Onset Computer Corporation, Bourne, MA) directly fixed on top of the container housing the PM_2.5_ monitor. The real-time temperature and relative humidity were recorded at 1-min intervals and averaged over 30-min segments corresponding to the time segments during which PM_2.5_ and arrhythmias were measured. The weather variables were treated as repeated measures, and each individual contributed 48 data points for each.

*Statistical analysis.* Two-sample *t*-tests, Wilcoxon-Mann-Whitney tests, chi-square tests, or Fisher’s exact tests, as appropriate, were used to compare the distributions of basic demographic variables between participants with and without chronic diseases. We used distributed lag models (Almon 1965; Pope and Schwartz 1996; Schwartz 2000) under the framework of a negative binomial regression model (Hilbe 2007) and generalized estimating equations (Hardin and Hilbe 2003) to assess the regression coefficients measuring the association between 30-min PM_2.5_ and each of the arrhythmic beat counts. Negative binomial regression can be viewed as an extension of Poisson regression, and, as for Poisson regression, the exponentiated regression coefficients represent rate ratios (RRs). However, unlike Poisson regression, negative binomial regression does not require the strong, and often violated, assumption that the variability of counts with a particular covariate pattern is equal to the mean. We specified a first-order autoregressive correlation structure to adjust for within-individual autocorrelation. In these models, one lag indicates a 30-min separation between the exposure and outcome. Thus, lag 0 indicates that the PM_2.5_ data were collected and averaged over the same 30-min segment as the ectopic beat count data, lag 1 indicates that the PM_2.5_ data were collected during the previous 30 min, lag 2 indicates that the PM_2.5_ data were collected during the 30-min segment 30–60 min before the ectopic beats were counted, and so forth. We chose a constrained distributed lag model, the polynomial distributed lag model, to reduce the potential collinearity of PM_2.5_ between individual lags using a second-degree polynomial. Because high levels of collinearity may exist for PM_2.5_ measurements taken closely together in time, the lag weights in the distributed lag model can be constrained to reduce collinearity by imposing a shape on the lag distribution, with a common approach being to constrain the lag weights to follow a low-order polynomial function. Thus, the model is termed a polynomial distributed lag model. Another advantage of the distributed lag model is its ability to provide interpretation of the cumulative effects of the lags included in the model, as well as individual lag effects. Cumulative effects are modeled by including all lags to be accumulated in a single model, and obtaining an estimate of the weighted average of the lag effects. Because PM_2.5_ exposures and ectopic beats were assessed in parallel over 48 lags (24 hr), we decided *a priori* to model no more than 10 lags, which allowed us to fit the distributed lag models using at least 80% of the data. Using PVC count per 30 min as the primary outcome variable, we started from the largest number of lags (lag 0–10) and reduced the total number of individual lags by back-eliminating the longer lags (e.g., lag 10), one lag at a time, until a significant cumulative effect from all the included lags (< 0.05) was identified (lag 0–1 for PVC in this article). We then used this model as our final model for all arrhythmia count variables. In addition to distributed lag models, we fitted moving average models that implicitly assume equal weighting of the lag effects, in contrast with the distributed lag model that allows the weighting to differ among the lags. We tested effect modification by sex and chronic disease status (hypertension, diabetes, or history of CVD) in the final model. The RR [95% confidence interval (CI)] for the PM_2.5_ and ectopy relationship at each level of the effect modifier was reported if there was significant effect modification (< 0.05). All results are expressed per 10-µg/m^3^ increase in PM_2.5_. We used SAS statistical software (version 9; SAS Institute Inc., Cary, NC) for all analyses.

## Results

**Figure 1:**
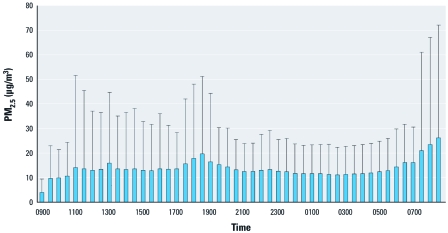
Thirty-minute segment-specific PM_2.5_ exposure (mean + SD) over 24 hr in the APACR study.

**Figure 2:**
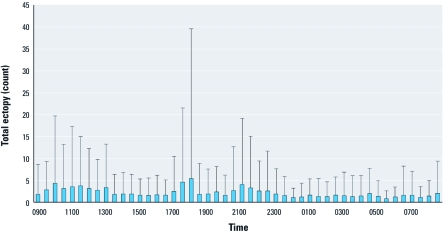
Thirty-minute segment-specific total ectopy count (mean + SD) over 24 hr in the APACR study.

Table 1 presents demographic and clinical characteristics and the ectopy counts in this community-based sample of 105 middle-age healthy nonsmokers. The mean age of the participants was 56 years, with 73% non-Hispanic white, 60% females, and 43% classified as having chronic disease (CVD, hypertension, or diabetes). The prevalence of CVD, hypertension, and diabetes was 7.6%, 35.2%, and 7.6%, respectively. At the population level, the 30-min mean ± SD PAC and PVC counts were 0.92 ± 4.94 and 1.22 ± 7.18, respectively. Participants classified as having chronic disease had significantly higher average PAC and PVC counts and significantly lower HRV indices (HF, LF, SDNN, and RMSSD) than did other participants (< 0.01 for all outcomes). The average (± SD) exposure to PM_2.5_ (over all 30-min segments) was 13.49 ± 22 μg/m^3^. The medians of the within-individual interquartile ranges (IQR) for the entire study sample on lag 0, lag 0–1, lag 0–2, lag 0–3, and lag 0–4 PM_2.5_ concentration were 6.38 μg/m^3^, 6.57 μg/m^3^, 5.97 μg/m^3^, 5.83 μg/m^3^, and 6.06 μg/m^3^, respectively, which indicates no substantial variation in the IQRs across the window of exposure. Therefore, we modeled the relationship between PM_2.5_ and ectopy risk per 10-μg/m^3^ increase of PM_2.5_ across all models. Figures 1 and 2 show the 30-min segment-specific distributions of the mean + SD of the PM_2.5_ levels and total ectopy counts (PVC and PAC counts combined) across the entire study population.

**Table 1:** Demographic characteristics of the study population.*a*

Table 1. Demographic characteristics of the study population.*a*								
		All subjects (*n* = 105)		Hypertension, diabetes, or CVD				
Characteristic				No (*n* = 60)		Yes (*n* = 45)		*p*-Value*b*
Age		56 ± 7.6		56 ± 8.2		57 ± 6.8		0.32
Sex (% male)		40		40		40		1.00
Race/ethnicity (% non-Hispanic white)		73		72		76		0.66
Glucose (mg/dL)		89 ± 25		85 ± 10		94 ± 36		0.10
BMI (kg/m^2^)		28 ± 5.9		26 ± 4.3		30 ± 7.1		< 0.01
CVD (%)		7.6		0.00		18		< 0.01
Hypertension (%)		35		0.00		85		< 0.01
Diabetes (%)		7.6		0.00		18		< 0.01
Blood pressure (mmHg)								
Systolic		122 ± 16		117 ± 12		128 ± 18		< 0.01
Diastolic		75 ± 9.2		73 ± 8.3		77 ± 9.8		0.02
Education, college or higher (%)		79		73		87		0.10
Medicine use (%)								
Hypertension		19		0.00		44		< 0.01
Diabetes		6.7		0.00		16		< 0.01
Arrhythmia		1.9		0.00		4.4		0.18
HRV indices								
Log HF (msec^2^)		6.70 ± 1.02		6.80 ± 1.01		6.56 ± 1.03		< 0.01
Log LF (msec^2^)		6.29 ± 0.96		6.41 ± 0.94		6.12 ± 0.96		< 0.01
SDNN (msec)		66.60 ± 31.32		69.37 ± 31.06		62.93 ± 31.29		< 0.01
RMSSD (msec)		25.76 ± 17.81		26.86 ± 17.98		24.30 ± 17.48		< 0.01
Ectopy variables								
PVC count		1.22 ± 7.18		0.91 ± 3.74		1.63 ± 10.05		< 0.01
PAC count		0.92 ± 4.94		0.66 ± 2.42		1.26 ± 6.99		< 0.01
Total ectopy count		2.14 ± 8.91		1.58 ± 4.51		2.90 ± 12.54		< 0.01
Lag 0 measures								
PM_2.5_ (μg/m^3^)		13.49 ± 21.60		11.88 ± 14.77		15.62 ± 28.06		< 0.01
Temperature (°C)		21.75 ± 3.54		21.84 ± 3.66		21.64 ± 3.38		0.05
Relative humidity (%)		39.65 ± 12.11		40.19 ± 12.32		38.93 ± 11.78		< 0.01
IQR*c* of PM_2.5_ (μg/m^3^)								
Lag 0		6.38		7.08		5.60		0.60
Lag 0–1		6.57		6.80		5.43		0.80
Lag 0–2		5.97		6.57		5.37		0.85
Lag 0–3		5.83		6.55		5.07		0.75
Lag 0–4		6.06		6.35		4.95		0.63
**a**Results are presented as mean ± SD for continuous variables and percentages for categorical variables. **b***p*-Value for comparing CVD and no-CVD groups. **c**The median of the within-individual IQRs of PM_2.5_ concentration.								

According to the model selection strategy described above, the final model for all outcomes contained only two lags: lag 0 (exposure and outcome measured during the same 30-min segment) and lag 1 (exposure measured during the previous 30 min). Table 2 presents the multivariable-adjusted RR (95% CI) for associations between PM_2.5_ (expressed in 10-µg/m^3^ increments) and PVC, PAC, and total ectopy counts. Associations of PM_2.5_ with PVC and total ectopy counts were statistically significant for lag 0 and lag 0–1 models. For example, the RR (95% CI) of PVC associated with a 10-μg/m^3^ increase in PM_2.5_ during lag 0 and lag 0–1 was 1.08 (1.05–1.10) and 1.03 (1.00–1.05), respectively, indicating an 8% and 3% increase in PVC counts, respectively. In contrast, PM_2.5_ was not positively associated with PAC count. The RRs (95% CI) from moving average exposure models (data not shown) are comparable to those from distributed lag models in Table 2. For comparison purpose, we also present in Table 3 estimated RRs (95% CI) from individual lags and cumulative RRs (95% CI) of PVC, PAC, and total ectopy counts from models that included all possible lags of PM_2.5_ exposures (lags 0–10). The individual lag RRs presented in Table 3 are consistent with the final models in Table 2: the very close individual lags (lag 0 and lag 1), but not the longer lag terms (lag 2 to lag 10), are significantly associated with higher RR of PVC and total ectopy counts, although some longer lag terms (lag 9 and lag 10) showed similar direction of their point estimates; and PM_2.5_ is not associated with PAC counts in these data.

**Table 2:** The cumulative RRs*a* (95% CIs) of ectopy counts associated with a 10-μg/m^3^ increment of PM_2.5_ concentration.

Table 2. The cumulative RRs*a* (95% CIs) of ectopy counts associated with a 10-μg/m^3^ increment of PM_2.5_ concentration.				
Ectopy variable		Lag		RR*b* (95% CI)
PVC count		Lag 0 (same time)		1.08 (1.05–1.10)**
		Lag 0–1 (30 min before)		1.03 (1.00–1.05)*
PAC count		Lag 0 (same time)		0.94 (0.85–1.04)
		Lag 0–1 (30 min before)		0.97 (0.89–1.05)
Total ectopy count		Lag 0 (same time)		1.05 (1.02–1.07)**
		Lag 0–1 (30 min before)		1.03 (1.01–1.05)**
**a**The RRs were calculated by exponentiating the regression coefficients from distributed lag negative binomial models. An RR of 1.05 indicates a 5% increase in ectopy count/30 min in association with a 10-µg/m^3^ increase in PM_2.5_ concentration. **b**Adjusted for age, sex, race, chronic disease status, and the same lag period temperature and relative humidity. *p < 0.05. **p < 0.01.				

**Table 3:** RRs*a* (95% CIs) of ectopy counts associated with a 10-μg/m^3^ increment of PM_2.5_ concentration from individual lags.

Table 3. RRs*a* (95% CIs) of ectopy counts associated with a 10-μg/m^3^ increment of PM_2.5_ concentration from individual lags.						
Lag		PVC		PAC		Total ectopy
Lag 0		1.02 (1.00–1.04)		1.00 (0.97–1.01)		1.02 (1.00–1.03)
Lag 1		1.00 (1.00–1.01)		0.99 (0.97–1.01)		1.00 (1.00–1.01)
Lag 2		0.99 (0.98–1.00)		0.98 (0.96–1.01)		0.99 (0.98–1.00)
Lag 3		0.98 (0.96–1.00)		0.98 (0.96–1.00)		0.98 (0.97–1.00)
Lag 4		0.98 (0.95–1.00)		0.98 (0.96–1.00)		0.98 (0.96–1.00)
Lag 5		0.98 (0.95–1.00)		0.98 (0.96–1.00)		0.98 (0.95–1.00)
Lag 6		0.98 (0.95–1.01)		0.98 (0.97–1.00)		0.98 (0.96–1.00)
Lag 7		0.99 (0.96–1.02)		0.99 (0.97–1.01)		0.98 (0.96–1.01)
Lag 8		1.00 (0.97–1.03)		0.99 (0.98–1.01)		0.99 (0.97–1.01)
Lag 9		1.02 (0.98–1.06)		1.00 (0.98–1.03)		1.00 (0.99–1.03)
Lag 10		1.04 (0.98–1.10)		1.01 (0.98–1.05)		1.02 (0.99–1.06)
Cumulative		0.98 (0.79–1.21)		0.90 (0.74–1.09)		0.93 (0.80–1.09)
**a**The RRs were calculated by exponentiating the regression coefficients from distributed lag negative binomial regression model, adjusted for age, sex, race, chronic disease status, and the same lag period temperature and relative humidity.						

To examine the influence of cardiac autonomic modulation on the relationship between PM_2.5_ exposure and PVC count, we modeled PM_2.5_ lag 0 and PVC adjusting for HRV variables one at a time, in addition to the covariates as presented in Table 4. In summary, the association between PM_2.5_ and PVC was reduced from an estimated 8% increase in PVC count with a 10-μg/m^3^ increase in exposure to a 3–7% increase after adjustment for HRV indices. We observed the largest difference with adjustment for RMSSD (RR = 1.03; 95% CI, 1.0–1.05), a marker of parasympathetic modulation.

**Table 4:** HRV-adjusted RRs*a* (95% CIs) of PVC counts associated with a 10-µg/m^3^ increment of PM_2.5_ concentration in lag 0.

Table 4. HRV-adjusted RRs*a* (95% CIs) of PVC counts associated with a 10-µg/m^3^ increment of PM_2.5_ concentration in lag 0.				
Adjustment		RR*b* (95% CI)		*p*-Value
HRV adjusted				
Log HF		1.06 (1.04–1.09)		< 0.01
Log LF		1.06 (1.04–1.09)		< 0.01
SDNN		1.07 (1.05–1.10)		< 0.01
RMSSD		1.03 (1.00–1.05)		0.08
Not HRV adjusted		1.08 (1.05–1.10)		< 0.01
**a**The RRs were calculated by exponentiating the regression coefficients from distributed lag negative binomial regression model. **b**Adjusted for age, sex, race, chronic disease status, and the same lag period temperature and relative humidity.				

Associations between PM_2.5_ on PVC and total ectopy counts were significantly stronger in participants without CVD-related comorbidity than in participants classified as having chronic disease (values for both interactions < 0.05; Table 5). Interactions were not significant between chronic disease and PM_2.5_ when PAC was the outcome, or between sex and PM_2.5_ for any of the outcomes at the ≤ 0.05 level.

**Table 5:** RRs*a* (95% CIs) of ectopy counts associated with a 10-µg/m^3^ increment of PM_2.5_ concentration by chronic disease status.

Table 5. RRs*a* (95% CIs) of ectopy counts associated with a 10-µg/m^3^ increment of PM_2.5_ concentration by chronic disease status.						
				RR*b* (95% CI)		
Ectopy variable		Lag		No chronic disease		With chronic disease
PVC count		Lag 0 (same time)		1.20 (1.09–1.33)*		0.97 (0.93–1.02)
		Lag 0–1 (30 min)		1.26 (1.07–1.48)*		0.88 (0.77–1.03)
PAC count		Lag 0 (same time)		0.98 (0.87–1.10)		0.87 (0.78–1.00)
		Lag 0–1 (30 min)		1.00 (0.89–1.16)		0.88 (0.70–1.11)
Total ectopy count		Lag 0 (same time)		1.13 (1.04–1.24)*		1.00 (0.98–1.02)
		Lag 0–1 (30 min)		1.16 (1.03–1.28)*		1.01 (0.99–1.03)
**a**The RRs were calculated by exponentiating the regression coefficients from distributed lag negative binomial regression models. **b**Adjusted for age, sex, race, temperature, and relative humidity. *p-Value for PM_2.5_ and chronic disease interaction < 0.05.						

## Discussion

The mechanisms responsible for the consistently reported association between fine PM exposure and CVD mortality and morbidity are not fully understood. Previous studies have suggested several possible underlying mechanisms, including cardiac autonomic impairment as measured by lower HRV (Creason et al. 2001; Gold et al. 2000; He et al. 2010; Liao et al. 1999, 2004; Pope et al. 1999) and effects on ventricular repolarization (Campen et al. 2006; Ghelfi et al. 2008; Henneberger et al. 2005; Liao et al. 2010; Lux and Pope 2009; Samet et al. 2009; Yue et al. 2007). Various studies, including patient-based population studies, panel studies, large population-based cohort studies, and controlled exposure studies, have indirectly suggested short-term PM effects on cardiac electrophysiology and outcomes (including HRV) (Elder et al. 2007; Liao et al. 1999, 2004; Park et al. 2005), ventricular repolarization (Lux and Pope 2009; Yue et al. 2007), T-wave alternans (Zanobetti et al. 2009), myocardium ischemia (Zhang et al. 2009), and arrhythmias (Berger et al. 2006; Dockery et al. 2005; Ebelt et al. 2005; Liao et al. 2009; Sarnat et al. 2006). The time course from PM exposure to effects on cardiac arrhythmia has not been investigated systematically in a middle-age community-dwelling sample of healthy nonsmokers. Cavallari et al. (2008) reported early- and later-phase HRV responses at 2 hr and 9–13 hr after exposure, respectively. Rich et al. (2005, 2006a, 2006b) also reported evidence of acute effects (1 hr) and longer cumulative effects (24-hr moving average) of elevated PM and gaseous concentrations on severe arrhythmias in ICD patients. We reported evidence of PM_2.5_ effects on HRV approximately 4–6 hr after exposure (He et al. 2010) and of PM_2.5_ effects on ventricular repolarization 3–4 hr after exposure (Liao et al. 2010). In the present study of 105 community-dwelling healthy nonsmokers, exposure to PM_2.5_ was associated with PVC, but not with PAC, consistent with our findings in the WHI population (Liao et al. 2009) and with findings reported by Sarnat et al. (2006) from a panel study of older adults.

More important, our data suggest that exposures to PM_2.5_ are associated with increased PVC frequency, with an approximately 8% increase in the number of PVCs per 30 min for each 10-μg/m^3^ increase in PM_2.5_ concentration during the same time period (*p* < 0.01). The association was reduced to approximately 3% for each 10-μg/m^3^ increase in PM_2.5_ concentration averaged over the same time period and the previous 30 min (*p* < 0.05). The association decreases progressively to the null when exposure is averaged over longer time periods, which suggests that the effect of PM_2.5_ exposure on the presence and frequency of PVC within a 24-hr time period lasts for approximately 60 min. Previous studies of the time course of PM effects on arrhythmia suggested effects within days of exposure (Liao et al. 2009; Peters et al. 2000), but these studies were based on daily-average exposures before ECG, in contrast with the present study. To our knowledge, this is the first study designed to have sufficient temporal resolution of both exposure and ECG variables, for example, on the 30-min basis of exposure, outcome, and covariables. This study design enabled us to examine the acute effect of PM_2.5_ exposure on PVC counts. Another major strength of this study is that we measured PM_2.5_ exposures, the ECG outcome data, and the covariables at the individual level on a real-time basis over 24 hr. With these individual-level data, we can adjust for individual-level time-varying and non-time-varying confounding factors. Together with our findings of similar acute effects of PM_2.5_ on HRV (He et al. 2010) and ventricular repolarization (Liao et al. 2010), our data provide evidence of acute effects of PM_2.5_ exposure on cardiac electrophysiological profiles. We found no association between PM_2.5_ exposures and PAC, consistent with the report from the WHI population (Liao et al. 2009). Thus, the association between PM_2.5_ exposures and the total ectopy counts, defined as the sum of PVC and PAC counts, appears to be driven by the association with PVC.

Mechanisms that would explain associations between increased arrhythmia frequency and PM_2.5_ exposure are unknown at this time. It is biologically plausible that exposures to PM_2.5_ directly reduce parasympathetic modulation and increase sympathetic modulation, resulting in a decreased threshold for arrhythmia. HRV is a reliable measure of cardiac autonomic modulation. Although some studies have not supported an association between PM and HRV (Peretz et al. 2008; Sullivan et al. 2005) and others have reported inverse associations (Magari et al. 2002; Riediker et al. 2004; Wheeler et al. 2006), most published studies have suggested that PM exposure is associated with lower HRV indices. In the present study, associations with PM_2.5_ were attenuated but still positive when we adjusted for HRV variables, especially RMSSD, consistent with an intermediate effect of PM exposure on autonomic modulation. However, adjustment for HRV parameters did not completely eliminate associations between PM_2.5_ and PVC, and other mechanisms might also explain associations.

In these data, the PM_2.5_ and PVC association was significantly stronger among persons without the CVD-related chronic conditions (CVD, hypertension, or diabetes) than in persons with such conditions, who showed little evidence of an association. This finding is different from previous studies that showed significant adverse effects of PM_2.5_ on arrhythmia among patients implanted with an ICD (Dockery et al. 2005; Peters et al. 2000; Rich et al. 2005). One potential explanation for this discrepancy is that relatively weak effects of PM_2.5_ may be difficult to detect in individuals at high risk of PVC from their chronic disease. Also, participants with CVD-related chronic conditions may be taking antihypertensive and heart rhythm control medications that may prevent or reduce the occurrence of PVC. However, the robust association between PM_2.5_ and PVC among persons without chronic CVD-related conditions suggests that PM effects on arrhythmia are not limited to individuals with chronic conditions.

This study has several limitations. First, our findings may not apply to smokers or persons with a recent acute cardiac event, because we excluded such persons from the study population. Second, most participants reported that they stayed indoors most of the time during the 24-hr study period, except when they had to travel by automobile, and they had limited exposure to second-hand cigarette smoke. Thus, participants had relatively low levels of exposure to PM_2.5_, and we cannot determine whether exposures at higher levels would result in similar associations. Third, it is well known that sympathetic nervous activity increases during physical activity, which could be related to higher PM_2.5_ exposure if it occurs outdoors and could also decrease the threshold of arrhythmia. However, although it is possible that the PM_2.5_ and ectopy association in this study was confounded by physical activity, the vast majority of our participants reported staying indoors 97% of the 24-hr study period. Fourth, we did not collect the ECG data under a controlled, supine-position setting, and short-term variation due to other factors that may affect ectopy cannot be fully accounted for in our analysis. However, it is not feasible to keep a healthy participant in a supine indoor position for 24 hr, and even if this were achieved, the results from such a study design would not be generalizable to a real-world situation.

## Conclusion

A 10-µg/m^3^ increase in PM_2.5_ exposure was associated with an immediate increase in arrhythmic heart beats, specifically PVC beats, among healthy individuals. The time course of the association suggested that effects were limited to within approximately 60 min of the PM_2.5_ exposure.
